# Face Mask Use and Chalazion Procedures Over 8 Years of US Medicare Data

**DOI:** 10.1111/jebm.70133

**Published:** 2026-03-31

**Authors:** Giacomo Visioli, Evan E. Afshin, Gayatri Bajaj, Ebby Elahi

**Affiliations:** ^1^ Department of Ophthalmology Icahn School of Medicine at Mount Sinai New York New York USA; ^2^ Department of Sense Organs Sapienza University of Rome Rome Italy; ^3^ Department of Ophthalmology SUNY Downstate Brooklyn New York USA; ^4^ School of Medicine Touro College of Osteopathic Medicine New York New York USA; ^5^ Department of Otolaryngology Icahn School of Medicine at Mount Sinai New York New York USA; ^6^ Department of Public Health Icahn School of Medicine at Mount Sinai New York New York USA; ^7^ Fifth Avenue Associates New York New York USA

The COVID‐19 pandemic had a heterogeneous impact across the United States, with multiple hotspots and peaks of cases occurring in different regions at different times. Moreover, individual states implemented varying mask mandates and public health policies, leading to widespread and prolonged face mask use and changes in healthcare access [1]. Although chalazion is a relatively minor condition, it remains a common reason for patients to seek ophthalmologic consultation, given its potential for discomfort, pain, recurrence, and, in cases of significant size, impairment of eyelid function [2]. During the pandemic, several clinical observations and retrospective studies suggested an increased incidence of chalazion, hypothesizing a potential link to prolonged face mask use [3, 4, 5]. Proposed mechanisms include altered periocular airflow promoting tear film evaporation [6], increased eyelid irritation and dehydration [7], and mechanical manipulation from frequent mask adjustments [8]. However, the available evidence remains largely circumstantial, based on small series, surveys, or limited regional datasets, without large‐scale confirmation [9]. Importantly, daily mask use was already a common practice in parts of Asia before the COVID‐19 pandemic, without evidence of increased chalazion incidence. This raises the question of whether the observed trend is attributable to pandemic‐related changes in healthcare‐seeking behavior or simply represents normal year‐to‐year variability. To address this issue on a broader scale, we analyzed national Medicare billing data from 2016 to 2023 to evaluate trends in chalazion‐related procedures and to assess whether the pandemic and widespread mask use influenced their frequency or management in the United States.

This study used publicly available, de‐identified data and was exempt from Institutional Review Board approval. Research adhered to the principles of the Declaration of Helsinki. We obtained publicly available Medicare Part B claims data (Centers for Medicare & Medicaid Services [CMS]) covering the years 2016 through 2023. The analysis focused on procedures billed under Current Procedural Terminology (CPT) codes 67700 (incision and drainage of an eyelid abscess, which may include inflamed or atypical chalazia) and 67800 (chalazion excision). Data were stratified by US state, and only states consistently reporting both CPT codes across all 8 years were included in the analysis (27 states). To account for differences in population size, annual procedure rates were calculated for each state and expressed as the number of procedures per 100,000 inhabitants using US 2020 Census Bureau population estimates. States were then stratified into two groups according to population size (≥10 million vs. <10 million inhabitants). Comparisons between these groups were performed annually using independent two‐sample *t*‐tests, and a one‐way analysis of variance (ANOVA) was used to assess whether combined annual procedure rates differed across years. We also calculated the ratio of billed services to unique patients for each CPT code as an indirect measure of repeat interventions. Medicare reimbursement amounts per procedure were reviewed to assess potential economic influences on coding and management patterns. All statistical analyses were performed using STATA version 18.0 (StataCorp LLC, College Station, TX, USA).

Analysis of Medicare claims data from 2016 to 2023 showed distinct trends in chalazion‐related procedures. CPT 67800 procedures (chalazion excision) increased until 2019, declined markedly in 2020, and remained lower in the following years. In contrast, CPT 67700 procedures (incision and drainage of eyelid abscess) showed a progressive increase throughout the study period without a decline during the pandemic years (Figure 1).

**FIGURE 1:**
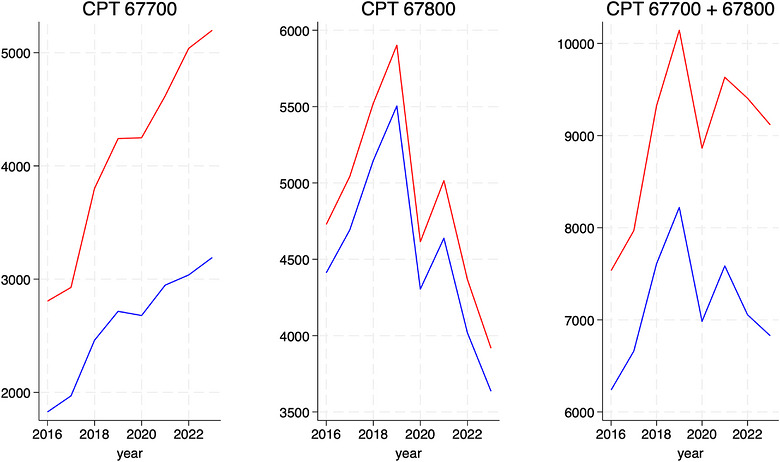
**Trends in chalazion‐related procedures before and after the COVID‐19 pandemic in the United States**. The three panels display the annual number of patients and billed services for (A) CPT codes 67700, (B) CPT codes 67800, and (C) the combined total of both procedures. The blue lines represent the number of unique patients undergoing each procedure, while the red lines indicate the total number of billed services.

The combined annual incidence per 100,000 inhabitants, summing CPT 67700 and CPT 67800, is reported in Table 1. When the two procedures were analyzed together, the overall annual procedure rate remained relatively stable across the study period despite year‐to‐year fluctuations. A one‐way ANOVA comparing the combined annual procedure rates across years did not show significant differences (*p* = 0.943). When stratified by population size, no significant differences were observed between states with ≥10 million inhabitants and those with smaller populations in any year (all *p* > 0.13).

**TABLE 1:** Annual rates of chalazion‐related procedures (CPT 67700 and 67800 combined) per 100,000 inhabitants in 27 US states from 2016 to 2023, stratified by state population size^*^

Year	Total Procedure rate (per 100,000)—27 states	Procedure rate (per 100,000)—10 states with Population ≥10 million	Procedure rate (per 100,000)—17 states with Population <10 million	*p* value
2016	2.50 ± 1.76	2.53 ± 1.80	2.48 ± 1.79	0.949
2017	2.39 ± 1.65	2.75 ± 1.92	2.19 ± 1.51	0.395
2018	2.91 ± 2.08	3.23 ± 2.75	2.72 ± 1.63	0.549
2019	3.01 ± 2.27	3.63 ± 3.08	2.65 ± 1.62	0.285
2020	2.53 ± 1.90	3.09 ± 2.84	2.20 ± 1.00	0.252
2021	2.59 ± 2.27	3.44 ± 3.18	2.09 ± 1.41	0.137
2022	2.44 ± 2.37	3.24 ± 3.42	1.99 ± 1.45	0.186
2023	2.51 ± 2.28	3.18 ± 3.38	2.17 ± 1.47	0.257

^*^Values are presented as mean ± standard deviation. *p*‐Values were calculated using independent two‐sample *t*‐tests comparing states with ≥10 million inhabitants and states with <10 million inhabitants for each year.

The ratio of billed services to unique patients remained stable over time. The mean ratio was 1.08 ± 0.01 for CPT 67800 and 1.57 ± 0.05 for CPT 67700. Regarding Medicare reimbursement, mean payment per procedure for CPT 67700 increased from $206.3 in 2016 to $225.9 in 2023. Similarly, CPT 67800 reimbursement slightly increased from $92.0 to $95.7 over the same period.

This analysis of Medicare data from 2016 to 2023 provides a comprehensive view of chalazion management trends in the United States during the COVID‐19 pandemic. Contrary to early hypotheses suggesting that widespread mask use might have increased the incidence of chalazion [3, 4, 5, 10], our findings do not demonstrate a sustained rise in chalazion‐related procedures at the population level. National‐level evidence therefore does not support the assumption that mask wearing contributed significantly to chalazion development.

Chalazion is often managed conservatively, particularly in its initial stages. Standard first‐line treatment usually consists of warm compresses applied several times daily, lid hygiene, and, in selected cases, topical therapy; oral antibiotics may be considered in specific settings such as associated rosacea or recurrent inflammatory disease. For lesions that persist despite conservative treatment, remain symptomatic, or are large enough to cause discomfort or visual disturbance, procedural management may be considered, including intralesional corticosteroid injection or incision and curettage [2]. This treatment spectrum is important for interpreting our findings, because Medicare procedural claims capture only lesions that underwent intervention and do not include chalazia managed conservatively.

The combined volume of CPT 67700 and CPT 67800 procedures remained relatively stable throughout the study period, suggesting that the observed variations between individual codes are more consistent with shifts in coding patterns than with substantial changes in disease incidence. Within this stable overall trend, CPT 67800 procedures (chalazion excision) declined markedly in 2020. This decrease may be partially explained by pandemic‐related disruptions in healthcare access, as elective procedures were widely postponed and many patients deferred non‐urgent care during the early phase of the COVID‐19 pandemic.

However, because the combined number of procedures remained broadly stable, the decline in CPT 67800 is unlikely to reflect a true reduction in chalazion requiring intervention. Instead, the concurrent increase in CPT 67700 procedures (incision and drainage of eyelid abscess) suggests a redistribution between procedure codes. In clinical practice, inflamed chalazia or atypical lesions may occasionally be coded as eyelid abscess drainage, particularly when diagnostic overlap exists between chalazion, hordeolum or pyogenic granuloma. Taken together, these findings are more consistent with shifts in coding practices or reimbursement dynamics than with a true epidemiologic change in chalazion occurrence.

These findings highlight key methodological issues in linking behavioral changes, such as mask use, to disease incidence. Observing two concurrent phenomena (e.g., an increase in mask wearing and anecdotal reports of more chalazia) does not establish causality [11]. The reports published during the pandemic provided early signals but lacked epidemiological confirmation. As emphasized in broader evaluations of research methodology, establishing causality requires more than temporal association; it requires accounting for confounding, recognizing access‐to‐care biases, and considering coding shifts over time [12].

Strengths of this study include the use of a large, standardized national dataset spanning 8 years, allowing evaluation of both short‐term and longitudinal trends. The consistent methodology across states strengthens internal validity. However, several limitations must be acknowledged. Medicare data represent an older population and may not capture trends in younger age groups. Patients outside the Medicare system, such as those with private insurance, are not included. Billing codes may also misclassify eyelid inflammatory lesions. While CPT 67800 specifically refers to chalazion excision, CPT 67700 describes incision and drainage of an eyelid abscess; in clinical practice, inflamed chalazia or hordeola may occasionally be coded under this category, creating potential overlap between diagnostic entities. Finally, the dataset provides no information on individual‐level mask use, compliance, or duration of exposure. Despite these constraints, substantial changes in disease incidence would be expected to influence national procedure volumes, which remained stable across the study period.

Despite initial concerns and small clinical series suggesting a link between mask use and chalazion, our analysis of Medicare data does not support this hypothesis. The combined rate of chalazion‐related procedures remained broadly stable throughout the study period. The divergent trends observed between CPT 67700 and CPT 67800 are therefore more consistent with shifts in coding practices or reimbursement dynamics than with a true epidemiologic effect of mask wearing. Although procedure rates reflect healthcare utilization patterns among Medicare beneficiaries and should not be interpreted as direct measures of chalazion incidence in the general population, large‐scale population data remain essential to critically reassess widely circulated clinical claims and to distinguish true epidemiologic signals from artefacts of healthcare delivery.

## Conflicts of Interest

The authors declare no conflicts of interest.
